# Evaluating Internet-Based AI Chatbots as Unregulated Digital Health Resources in Dental Emergency Management: Comparative Multilingual Study

**DOI:** 10.2196/96731

**Published:** 2026-10-27

**Authors:** Ahmed Bashah, Quan Huixin, Hamza Zaawari, Ali Al-Waqeerah, Eslam Ghaleb, Abdulkhaleq Salem, Natheer Wahan, Gang Chen

**Affiliations:** 1 Department of Stomatology, The First Affiliated Hospital of Dalian Medical University, No.222, Zhongshan Road, Dalian, Liaoning, 116011, China, +86 18098873280; 2 College of Dentistry, Hodeidah University, Al Hudaydah, Yemen; 3 Department of Respiratory, The First Affiliated Hospital of Dalian Medical University, Dalian, Liaoning, China; 4 Department of Biochemistry and Molecular Biology, Dalian Medical University, Dalian, Liaoning, China; 5 Department of Pharmacology, Dalian Medical University, Dalian, Liaoning, China

**Keywords:** artificial intelligence, large language models, chatbots, dental medical emergencies, multilingual evaluation, prompt engineering

## Abstract

**Background:**

Publicly accessible, internet-based AI chatbots have emerged as unregulated digital health resources that clinicians and patients consult in real time, including during acute medical emergencies. In dental practice—where life-threatening events such as anaphylaxis, cardiac arrest, and severe hypoglycemia can unfold within seconds—the unsupervised use of these tools as point-of-care decision aids poses uncharacterized patient safety risks. Despite widespread deployment, no study has systematically evaluated the accuracy, temporal consistency, or multilingual reliability of contemporary large language model (LLM)–based chatbots specifically for dental medical emergency management.

**Objective:**

This study aimed to evaluate the accuracy, temporal stability, and multilingual performance of five contemporary LLM-based chatbots when queried on the management of medical emergencies in dental practice.

**Methods:**

A comparative study was conducted to assess five AI chatbots: ChatGPT-5.2 (OpenAI), Gemini 3.1 (Google), DeepSeek-R1 (DeepSeek AI), Grok 4.20 (xAI), and Kimi K2.5 (Moonshot AI). A total of 130 questions covering dental medical emergency management were developed and categorized into four formats: multiple-choice questions (MCQs), yes-or-no questions, open-ended WH questions, and clinical scenario questions. Each question was presented using two prompting strategies (basic and advanced) across three languages (English, Arabic, and Chinese). Data collection was performed over seven consecutive days, generating 27,300 chatbot responses. Responses were evaluated by language-specific expert reviewers and classified as correct or incorrect based on established clinical references. Statistical analysis was performed using chi-square tests to assess associations between accuracy and study variables. Pairwise post hoc comparisons of column proportions were conducted using z-tests with Bonferroni correction.

**Results:**

A total of 27,300 responses were analyzed; 25,377 (92.96%) were correct. Question type significantly influenced chatbot performance (*P*<.001), with MCQs demonstrating the highest accuracy (5942/6300, 94.3%), and WH questions demonstrating the lowest accuracy (7686/8400, 91.5%). Language also showed a significant association with accuracy (*P*=.01), with English prompts achieving the highest accuracy (8512/9100, 93.5%). Chatbot model performance differed significantly (*P*<.001), with Gemini 3.1 demonstrating the highest accuracy (5176/5460, 94.8%; 95% CI 94.21%‐95.39%), followed by ChatGPT-5.2 (5105/5460, 93.5%; 95% CI 92.85%‐94.15%) and Kimi K2.5 (5071/5460, 92.9%). Prompt complexity and testing day did not significantly affect response accuracy (*P*=.69 and *P*=.96, respectively).

**Conclusions:**

Current LLM-based chatbots demonstrate high accuracy in answering structured questions related to dental medical emergency management. However, performance varies according to question format and language, and the presence of incorrect responses highlights potential safety concerns. AI chatbots may serve as educational tools, but their use as real-time decision support during dental medical emergencies should be approached with caution.

## Introduction

AI has rapidly emerged as a transformative technology across multiple scientific and medical disciplines. AI is broadly defined as the simulation of human intelligence processes by computer systems. It can analyze complex datasets and perform tasks that typically require human intelligence, such as pattern recognition, prediction, and decision-making. In health care, AI technologies are increasingly used to analyze clinical information, assist in diagnosis, support treatment planning, and enhance patient management [1]. They are also regarded as powerful and disruptive technologies that can fundamentally transform clinical practice [2]. A core subset of AI is machine learning (ML), which uses algorithms to learn patterns from data and make predictions or decisions [3]. ML systems are typically categorized into generative and discriminative approaches [4]:discriminative models, which focus on classification or prediction, and generative models, which are capable of producing new data outputs.

Among generative approaches, large language models (LLMs) have attracted considerable attention for their ability to generate natural language responses from large-scale training datasets. LLMs operate using deep neural networks that learn linguistic patterns and contextual relationships from large text corpora [5]. LLMs are a specialized type of generative AI (GenAI) designed to generate natural-language text [6]. They are typically implemented as transformer-based neural networks trained on large-scale text corpora from the internet and other sources. They are rapidly transforming various domains, including biomedicine and health care [6]. In the biomedical and health domains, LLMs have demonstrated strong capabilities in understanding clinical language and generating coherent medical text [7]. They can process unstructured clinical documents, answer medical questions, and assist in drafting clinical notes or summaries. Therefore, LLMs are increasingly viewed as general-purpose engines for language-centric tasks in health care information systems [6,8].

Researchers and medical professionals are seeking ways to integrate LLMs into diagnostic support and clinical administrative tasks [9]. Regardless, actions involving LLMs could pose a risk of compromising both individual and public health due to the potential for judgment error [10]. The capabilities of LLMs are commonly delivered to users through conversational interfaces known as GenAI chatbots. These systems translate the underlying language modeling capabilities into interactive dialogue platforms that can answer questions, generate explanations, and assist with information retrieval [11].

Chatbots are AI-driven software systems designed to simulate human dialogue via text or speech, delivering natural-language responses to user inquiries in a conversational manner. Every AI chatbot uses a distinct source of information to search the internet and is designed to create new content, including text, in response to user prompts [12]. Advanced AI chatbots include Gemini (Google), ChatGPT (OpenAI), DeepSeek (DeepSeek AI), Grok (xAI), and Kimi (Moonshot AI). Among these systems, high accuracy has been achieved. However, these models can still produce incorrect or fabricated information, a phenomenon often referred to as hallucination [13]. Newer chatbots, including proprietary LLM systems, are being adapted for clinical communication, but comprehensive safety assessments remain limited [14].

AI has rapidly emerged as a transformative force in medicine across diagnostics, treatment, and patient care [15]. AI tools can improve diagnostic accuracy and efficiency by analyzing complex medical data that exceeds human processing capacity [2]. Currently, clinical decision support and medical imaging analysis are among the most common applications of AI in health care [16]. Despite these advances, there are concerns about the bias, transparency, and reproducibility of AI models in real clinical settings [17]. Ethical analyses highlight unresolved issues regarding privacy, fairness, and accountability when AI is used in medical decision-making [18]. Legal scholars emphasize that current liability frameworks are not yet fully prepared to allocate responsibility for AI-related medical errors [19]. Dentistry has recently begun to adopt AI systems for analyzing clinical dental data and supporting diagnosis [20,21]. AI algorithms, particularly deep learning models, have demonstrated high performance in detecting dental diseases on radiographic images [22]. These systems have been applied across multiple dental subspecialties, including radiology, periodontics, orthodontics, and prosthodontics [22]. Studies indicate that AI can achieve diagnostic accuracy comparable to or better than that of less-experienced dentists in periodontal assessment. The use of AI in dentistry is expected to reduce treatment time and clinician workload while maintaining or improving diagnostic quality [15]. However, authors consistently note that most AI tools in dentistry still require further validation in real-world clinical settings [20].

A medical emergency in the dental office is commonly described as an acute situation that poses an immediate risk to life or long-term health [23]. Medical emergencies in the dental practice, although relatively infrequent, represent highly critical events that can lead to severe morbidity or patient mortality if not managed with immediate and precise intervention [24]. Common emergencies ranging from vasovagal syncope and hypoglycemia to life-threatening conditions such as anaphylaxis, myocardial infarction, and cardiac arrest require the dental practitioner to act swiftly as a first responder [25]. This gap in clinical readiness is frequently attributed to a lack of intensive, simulation-based emergency training and to insufficient real-world exposure during undergraduate dental education [26]. Faced with a high-stress clinical emergency and lacking the requisite practical experience, inexperienced practitioners may instinctively seek rapid external assistance. Recently, the exponential rise in the popularity and accessibility of AI chatbots and LLMs has positioned these tools as instantaneous sources of medical information [27].

Because LLMs can generate highly articulate, rapid responses to user queries, there is a growing yet alarming potential for distressed clinicians to use these conversational agents as real-time consultative tools during an unfolding medical crisis in the dental chair [28,29]. While AI has demonstrated utility in offline medical education and drafting clinical text, its application as an active decision-support tool in acute, life-threatening scenarios introduces unprecedented risks. LLMs are fundamentally predictive text models that lack genuine clinical reasoning, situational awareness, or the ability to assess real-time vital signs [6]. Consequently, they are prone to hallucinations—generating responses that appear highly confident and structurally sound but are factually incorrect, contextually inappropriate, or dangerously incomplete [30]. In the context of a dental medical emergency, where intervention windows are measured in seconds, relying on AI-generated advice is highly hazardous. Errors in algorithmic outputs regarding critical drug dosages (eg, epinephrine in anaphylaxis) or the correct sequence of basic life support (BLS) protocols can waste critical time and lead to catastrophic clinical outcomes, including irreversible neurological damage or patient death [8]. Therefore, evaluating the actual clinical accuracy, safety, and reliability of these AI models when presented with acute dental emergency scenarios is of paramount importance to prevent their misuse and establish clear boundaries for AI application in clinical dentistry [31,32]. Because clinicians and laypeople now routinely turn to publicly accessible AI chatbots for urgent, high-stakes health information outside any regulated or supervised setting, the dental medical emergency, serves as a clinically concrete test case for a much wider public health question—the reliability of consumer-facing GenAI as an unsupervised source of time-critical medical guidance.

General dental practitioners can expect to encounter medical emergencies with a frequency of approximately one event every few years. Vasovagal syncope is reported as the most frequent emergency, followed by conditions such as angina, hypoglycemia, and seizures. Effective management of these events depends on rapid recognition, initial stabilization, and the prompt activation of emergency medical services. Professional organizations emphasize that all members of the dental team must be trained to respond to emergencies with clear protocols. Because of the time-critical nature of these events, any delay or error in decision-making may have serious consequences for patient safety [33].

In acute and emergency care medicine, AI-based tools are being investigated for triage, risk prediction, and early detection of clinical deterioration [3]. These systems aim to support rapid decision-making by synthesizing large volumes of physiological and clinical data in real time [34]. Despite this interest, most existing AI applications in dentistry focus on planned diagnostic or treatment tasks rather than on acute emergencies [20]. There is very limited evidence on the performance of AI tools for guiding the management of medical emergencies in dental settings [22]. LLM-based chatbots could, in principle, offer stepwise guidance on differential diagnosis and emergency drug protocols during dental emergencies [7]. However, the risk of hallucinated or incomplete recommendations in time-critical scenarios raises serious concerns about diagnostic error and patient harm [13]. Current evaluations of AI in medicine largely examine performance on retrospective datasets, simulations, or written examinations rather than real-time emergencies [35]. From a digital health informatics perspective, AI chatbots represent a disruptive category of consumer-facing health technology whose adoption has outpaced evaluation. Studies published in major digital health journals have examined LLM performance across medical licensing examinations and specialty questions; however, none have specifically addressed the medical emergencies in the dental office, nor have any compared performance across multiple languages in a single experimental design. The multilingual dimension is especially relevant to digital health equity, as non–English speaking clinicians and patients face additional barriers when accessing reliable AI-generated health information. The present study addresses these gaps by providing the first large-scale, multilingual, multiplatform evaluation of LLM chatbot accuracy in dental emergency decision support. Most dental AI research has not specifically addressed medical emergencies occurring in the dental office. This study aims to evaluate the performance of 5 commercially available AI chatbots in answering questions related to medical emergency management in dental practice. The primary objective was to assess the accuracy, consistency, and reliability of AI-generated responses across different question formats, prompt complexity levels, testing periods, and language contexts.

## Methods

### Study Design

A systematic evaluation of 5 AI chatbots was conducted over 7 days (March 1‐7, 2026) to assess their accuracy in addressing clinically relevant queries on medical emergency management in the dental practice. Four types of questions—multiple-choice questions (MCQs), yes or no, open-ended WH questions, and clinical scenario questions—were used across two different levels of promotion: basic and advanced prompts, among three languages: English, Arabic, and Chinese. A total of 130 questions were designed based on the clinical guidelines [33,36-39], as well as the evolve instructor resources accompanying Malamed’s Medical Emergencies in the Dental Office, Seventh Edition [24] (Figure 1). All 5 platforms were evaluated in their publicly accessible, free-tier configurations, consistent with the real-world conditions under which clinicians without institutional AI access would consult these tools.

**Figure 1.**
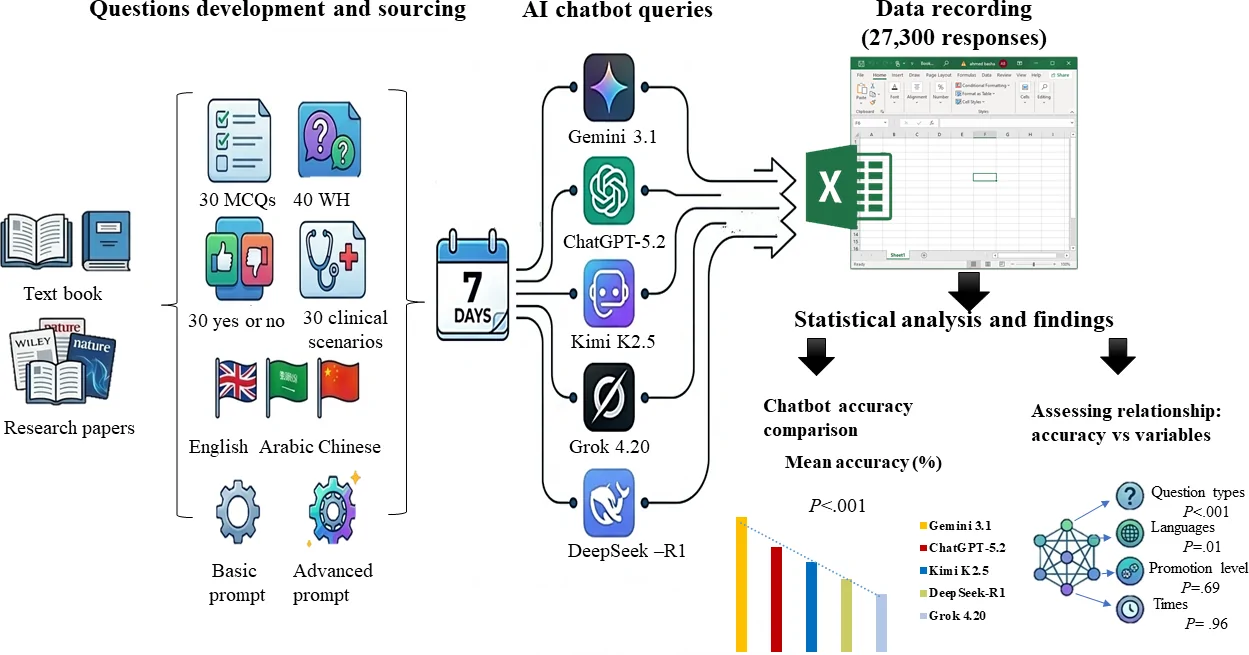
Graphical abstract of the study design. MCQ: multiple-choice question.

### Question Development and Categorization

A total of 130 questions, 30 MCQ, 30 yes or no questions, 40 WH open-ended questions, and 30 clinical scenario questions focused on knowledge, diagnostic criteria, and management protocols of medical emergency management in the dental practice (Multimedia Appendix 1). All questions were designed to meet predefined criteria: clinical relevance, practical applicability, and comprehensive coverage of medical emergency management, from diagnosis through management. Questions were designed to reflect real-world clinical scenarios encountered in dental practice and covered critical topics, including cardiovascular emergencies, respiratory complications, allergic reactions, and emergency medication protocols. This distribution was designed to comprehensively assess the performance of 5 chatbots across varying levels of question complexity and promotion, across testing periods, and language contexts.

### Multilingual Question Development and Translation

To evaluate chatbot performance across linguistic contexts, all 130 questions were originally developed in English and subsequently translated into Arabic and Simplified Chinese. Each question was independently translated from English to Arabic and Chinese by two dental professionals who were native speakers of the target language and fluent in English. All translators possessed expertise in dental emergency medicine and familiarity with medical terminology in both languages. Both basic and advanced prompts were culturally adapted for each language while maintaining conceptual equivalence, resulting in a comprehensive trilingual question bank comprising 390 questions (130 per language).

### Prompt Engineering Strategy

To assess how prompt sophistication affects chatbot accuracy, two prompting strategies were developed. The first is basic prompts: questions asked in simple, direct language without extra context or instructions. The second is advanced prompts: questions asked with fixed instructions that were added before each question. The instruction was applied the same way for all chatbots and all questions:

This is a question about medical emergency management in a dental clinic. You are an expert dental professional. Base your answer strictly on real, verifiable medical information retrieved from peer-reviewed literature and established clinical guidelines. Do not generate speculative or fabricated content, as an incorrect answer could result in patient harm.

The purpose of this instruction was to check whether telling the model to rely on evidence-based, literature-grounded reasoning improves its accuracy in questions supplemented with contextual information and clear instructions on response format, compared with the basic prompt. Each of the 130 questions was crafted in both basic and advanced formats across all three languages, enabling a direct comparison of how prompt complexity affects response accuracy within and across different linguistic contexts.

### AI Chatbot Selection

Five AI chatbots were selected for evaluation based on their widespread availability, distinct architectures, and popularity among health care professionals: ChatGPT-5.2, Gemini 3.1, DeepSeek-R1, Grok 4.20, and Kimi K2.5. These models represent various large language model architectures and training methods, providing a comprehensive assessment of current AI capabilities in medical domains. The following AI platforms were evaluated for their advanced analytical features.

- ChatGPT-5.2: free version and Applied Research feature for nuanced responses.
- DeepSeek-R1: DeepThink and the search feature.
- Grok 4.20: Operated in Expert (Think Hard) enabled.
- Google Gemini: Access to the Gemini 3.1 model.
- Kimi K2.5: K2.5 Thinking model.

To simulate real-world use, all chatbots were accessed through their official public web interfaces.

### Data Collection Protocol

Data collection was conducted over seven consecutive days to assess temporal consistency and identify potential learning effects or performance degradation over time. Each question was submitted to all 5 chatbots using both basic and advanced prompts in all three languages on each testing day, generating a comprehensive dataset of 27,300 individual responses (130 questions×2 prompt types×5 chatbots×7 days×3 languages). Responses were recorded in real time within a structured Microsoft Excel spreadsheet. MCQ and yes or no questions, open-ended questions, and clinical scenario questions are coded as correct or incorrect based on the sources from which the questions were extracted. To ensure standardization and minimize bias, the following data collection procedures were implemented. Each chatbot interaction was conducted in a fresh session (New Chat) to prevent contextual carryover from previous queries; additionally, each session was conducted entirely in one language, with appropriate language settings configured for each chatbot. English queries were submitted first, followed by Arabic and Chinese.

### Response Evaluation and Scoring

All chatbot responses were evaluated by a language-specific expert, each consisting of three independent reviewers with expertise in dental emergency medicine and native fluency in the evaluation language: English, Arabic, and Chinese. Each researcher evaluated responses in one language. To determine whether a chatbot’s answer to a subjective clinical question was “correct,” each response was compared verbatim with the reference sources. For example, if the response to Q1 in the chatbot was “yes,” but the reference sources indicated “no” the response was classified as incorrect. In summary, responses that matched the reference sources were categorized as correct, and responses that did not match were categorized as incorrect. Chatbot model was assessed by overall response accuracy (percentage of correct responses), stratified by chatbot platform.

### Statistical Analysis

The data were analyzed using R (version 4.5.0; R Core Team) and SPSS (version 24; IBM Corp). Descriptive statistics included calculating the mean accuracy rates and 95% CI for each chatbot’s overall accuracy. Comparative analyses involved using Pearson chi-square test to evaluate the association between chatbot platforms and response accuracy, as well as examining relationships between categorical variables (question type, chatbot model, prompt type, testing day, and language) and response accuracy. Following each significant omnibus chi-square test, pairwise post hoc comparisons of column proportions were conducted using z-tests with Bonferroni-adjusted *P* values. Statistical significance was determined using the *P* value threshold at a level of .05.

### Ethical Considerations

This study did not involve human participants, patient data, or human biological material. All questions were developed for research purposes and contained no identifiable patient information. Therefore, ethical approval and informed consent were not required.

## Results

### Distribution of Chatbot Response Accuracy Across Study Variables

Table 1 shows the distribution of chatbot response accuracy across five primary study variables: question type, prompt complexity, chatbot model, testing day, and language. The analysis encompassed a total of 27,300 responses; 25,377 (92.96%) were correct, while 1923 (7.0%) were incorrect, as shown in Figure 2. Question type demonstrated a highly significant association with response accuracy (*P*<.001). MCQs yielded the highest accuracy rate at 5942 of 6300 (94.3%) correct responses, followed closely by yes or no questions at 5933 of 6300 (94.2%), and clinical scenarios at 5816 of 6300 (92.3%). WH questions showed the lowest accuracy at 7686 of 8400 (91.5%), with 714 of 8400 (8.5%) incorrect responses, accuracy was significantly lower than MCQs and yes or no questions. Language also emerged as a significant predictor of performance (*P*=.01). English prompts achieved the highest accuracy at 8512 of 9100 (93.5%), compared to 8456 of 9100 (92.9%) for Chinese and 8409 of 9100 (92.4%) for Arabic. Chatbot model was a highly significant predictor of accuracy (*P*<.001). Gemini 3.1 demonstrated superior performance with an accuracy rate of 5176 of 5460 (94.8%), followed by ChatGPT-5.2 at 5105 of 5460 (93.5%), and Kimi K2.5 at 5071 of 5460 (92.9%). DeepSeek-R1 and Grok 4.20 followed with 5030 of 5460 (92.1%) and 4995 of 5460 (91.5%), respectively. The error rate for Gemini 3.1 was 284 of 5460 (5.2%), which was notably lower than that of Grok with 465 of 5460 responses (8.5%), highlighting meaningful variability in model capabilities. In contrast to the variables above, prompt complexity (basic vs advanced) showed no significant effect on accuracy (*P*=.69). Advanced prompts resulted in 12,697 of 13,650 (93%) accuracy, while basic prompts yielded 12,680 of 13,650 (92.9%) accuracy. Similarly, the testing day showed no significant impact on performance (*P*=.96). Accuracy rates remained remarkably stable throughout the study period, ranging from 3617 of 3900 (92.7%) and 3615 of 3900 (92.7%) on the first and second days to 3634 of 3900 (93.2%) on the sixth and seventh days. This stability indicates a lack of performance degradation or day-to-day variability, reflecting consistent system reliability over time.

**Table 1.** Distribution of chatbot response accuracy across study variables. (N=27,300).

Variable and level	Correct, n (%)	Incorrect, n (%)	*P* value
Type of question			<.001
Clinical scenario	5816 (92.3)	484 (7.7)	
MCQ^a^	5942 (94.3)	358 (5.7)	
WH Question	7686 (91.5)	714 (8.5)	
Yes or no	5933 (94.2)	367 (5.8)	
Prompt			.69
Advanced	12,697 (93)	953 (7)	
Basic	12,680 (92.9)	970 (7.1)	
Chatbots			<.001
ChatGPT-5.2	5105 (93.5)	355 (6.5)	
DeepSeek-R1	5030 (92.1)	430 (7.9)	
Gemini3.1	5176 (94.8)	284 (5.2)	
Grok 4.20	4995 (91.5)	465 (8.5)	
Kimi K2.5	5071 (92.9)	389 (7.1)	
Days			.96
First day	3617 (92.7)	283 (7.3)	
Second day	3615 (92.7)	285 (7.3)	
Third day	3619 (92.8)	281 (7.2)	
Fourth day	3630 (93.1)	270 (6.9)	
Fifth day	3628 (93)	272 (7)	
Sixth day	3634 (93.2)	266 (6.8)	
Seventh day	3634 (93.2)	266 (6.8)	
Language			.01
Arabic	8409 (92.4)	691 (7.6)	
Chinese	8456 (92.9)	644 (7.1)	
English	8512 (93.5)	588 (6.5)	

a MCQ: multiple-choice questions.

**Figure 2.**
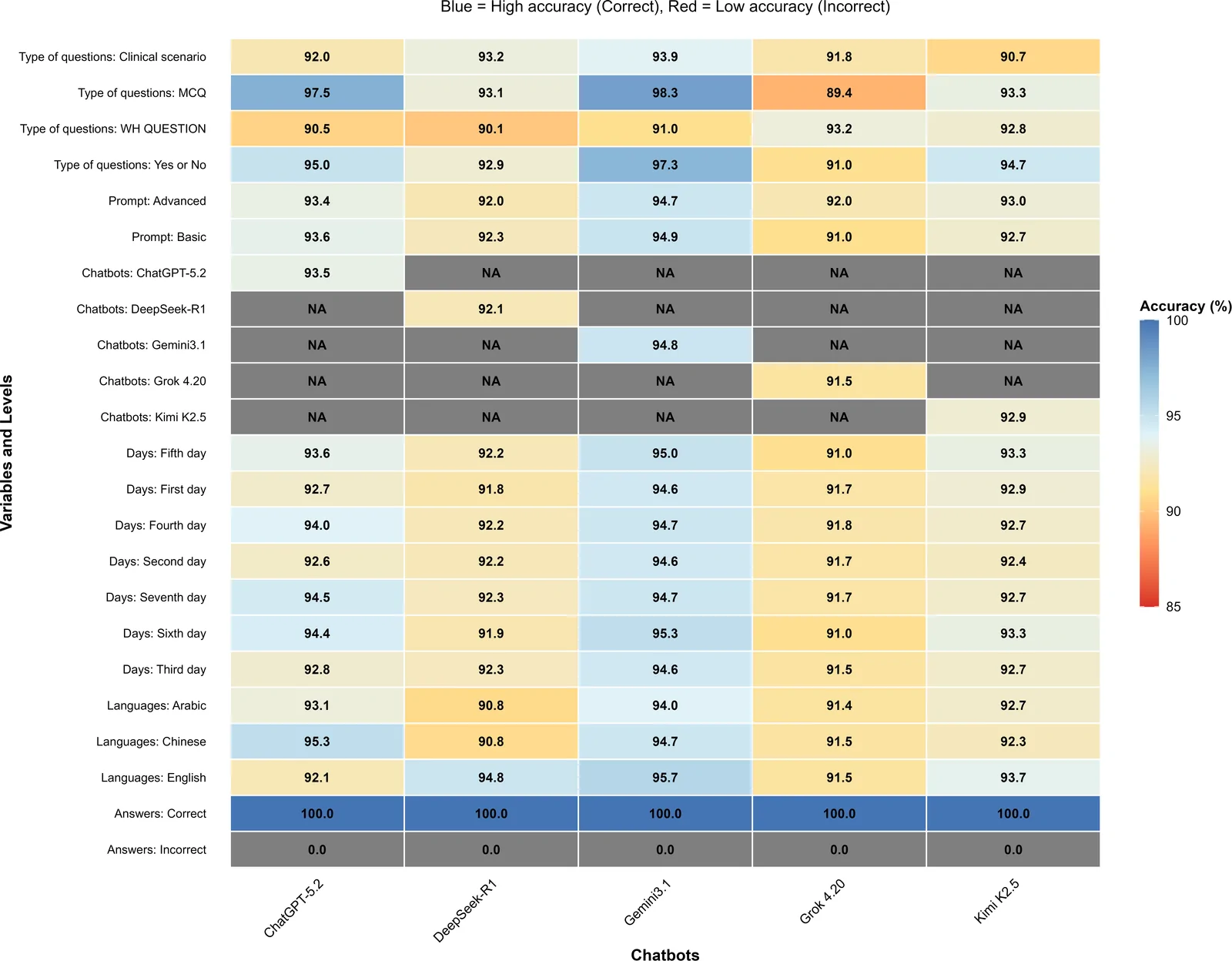
Heatmap illustrating the distribution of chatbot response accuracy across study variables. MCQ: multiple-choice question.

### Assessment of Stability and Reliability of Chatbot Performance Over Days

Table 2 illustrates the performance of the five chatbots across the 7-day study period, demonstrating high temporal stability across all evaluated chatbot models. Statistical analysis using chi-square tests indicated no significant variation in accuracy rates over time for any individual chatbot, with all *P* values significantly exceeding the .05 threshold. Gemini 3.1 maintained the most consistent high-level performance, with 743 of 780 (95.3%) on the sixth day (*P*>.99). DeepSeek-R1 and Kimi K2.5 similarly exhibited remarkable reliability, with accuracy rates for DeepSeek-R1 consistently remaining at or above 91.8% (*P*>.99) and Kimi K2.5 staying at or above 92.4% (*P*=.99) throughout the week. While ChatGPT-5.2 and Grok 4.20 showed minor daily fluctuations, their performance patterns remained statistically nonsignificant, with *P* values of .54 and .99, respectively. Figure 3 shows the distribution of response accuracy of chatbots over days. These findings confirm that the timing of assessment did not affect response accuracy, suggesting that the evaluated AI models yield stable clinical outputs that are not subject to significant day-to-day variability or performance degradation.

**Table 2.** Performance of chatbots across days.(N=780).

Chatbot and status	First day	Second day	Third day	Fourth day	Fifth day	Sixth day	Seventh day	*P* value
ChatGPT-5.2								.54
Correct, n (%)	723 (92.7)	722 (92.6)	724 (92.8)	733 (94.0)	730 (93.6)	736 (94.4)	737 (94.5)	
Incorrect, n (%)	57 (7)	58 (7)	56 (7)	47 (6)	50 (6)	44 (6)	43 (6)	
DeepSeek-R1								>.99
Correct, n (%)	716 (91.8)	719 (92.2)	720 (92.3)	719 (92.2)	719 (92.2)	717 (91.9)	720 (92.3)	
Incorrect, n (%)	64 (8)	61 (8)	60 (8)	61 (8)	61 (8)	63 (8)	60 (8)	
Gemini3.1								>.99
Correct, n (%)	738 (94.6)	738 (94.6)	738 (94.6)	739 (94.7)	741 (95.0)	743 (95.3)	739 (94.7)	
Incorrect, n (%)	42 (5)	42 (5)	42 (5)	41 (5)	39 (5)	37 (5)	41 (5)	
Grok 4.20								>.99
Correct, n (%)	715 (91.7)	715 (91.7)	714 (91.5)	716 (91.8)	710 (91.0)	710 (91.0)	715 (91.7)	
Incorrect, n (%)	65 (8)	65 (8)	66 (9)	64 (8)	70 (9)	70 (9)	65 (8)	
Kimi K2.5								.99
Correct, n (%)	725 (92.9)	721 (92.4)	723 (92.7)	723 (92.7)	728 (93.3)	728 (93.3)	723 (92.7)	
Incorrect, n (%)	55 (7)	59 (8)	57 (7)	57 (7)	52 (7)	52 (7)	57 (7)	

**Figure 3.**
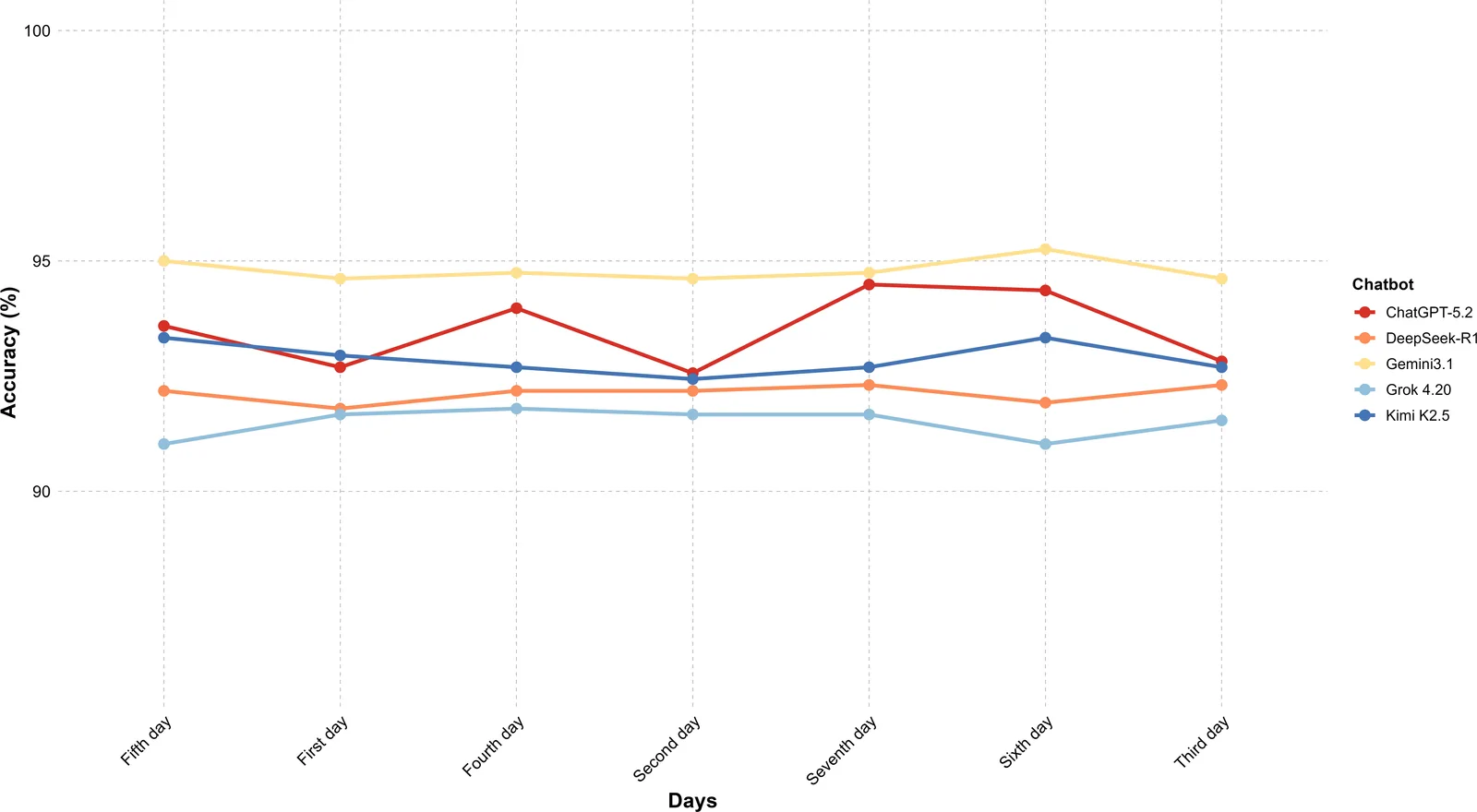
Distribution of chatbot accuracy over time.

### Impact of Prompt Level on Chatbot Performance

Table 3 shows the performance of the evaluated chatbots across the prompt levels advanced and basic prompts. Statistical analysis demonstrates that for all chatbot models tested, the prompt level did not yield a statistically significant difference in accuracy, with all *P* values exceeding the .05 threshold. Gemini 3.1 demonstrated the highest overall consistency and accuracy, maintaining a correct response rate 2586 of 2730 (94.7%) responses at the advanced level and (2590/2730, 94.9%) at the basic level (*P*=.81). Similarly, ChatGPT-5.2 exhibited high stability, with correct responses recorded at (2550/2730, 93.4%) and (2555/2730, 93.6%), respectively (*P*=.78). DeepSeek-R1 and Kimi K2.5 also maintained stable reliability across levels, with accuracy rates consistently remaining at or above (DeepSeek-R1: 2511/2730, 92.0% advanced; 2519/2730, 92.3% basic; Kimi K2.5: 2539/2730, 93.0% advanced, 2532/2730,92.7% basic; *P*=.69 and *P*=.71, respectively). Although Grok 4.20 showed a slight numerical decrease in performance at the basic level (2484/2730, 91.0%) compared to the advanced level (2511/2730, 92.0%), this shift did not reach statistical significance (*P*=.19). These results suggest that AI chatbots are highly resilient to changes in prompting strategies (Figure 4). The lack of significant variance across models indicates that the level of promotional complexity does not meaningfully impact the reliability of these chatbots in clinical knowledge assessment.

**Table 3.** Chatbot performance by prompt level. (N=2730).

Chatbot and status	Advanced, n (%)	Basic, n (%)	*P* value
ChatGPT-5.2			.78
Correct	2550 (93.4)	2555 (93.6)	
Incorrect	180 (6.6)	175 (6.4)	
DeepSeek-R1			.69
Correct	2511 (92)	2519 (92.3)	
Incorrect	219 (8)	211 (7.7)	
Gemini 3.1			.81
Correct	2586 (94.7)	2590 (94.9)	
Incorrect	144 (5.3)	140 (5.1)	
Grok 4.20			.19
Correct	2511 (92)	2484 (91)	
Incorrect	219 (8)	246 (9)	
Kimi K2.5			.71
Correct	2539 (93)	2532 (92.7)	
Incorrect	191 (7)	198 (7.3)	

**Figure 4.**
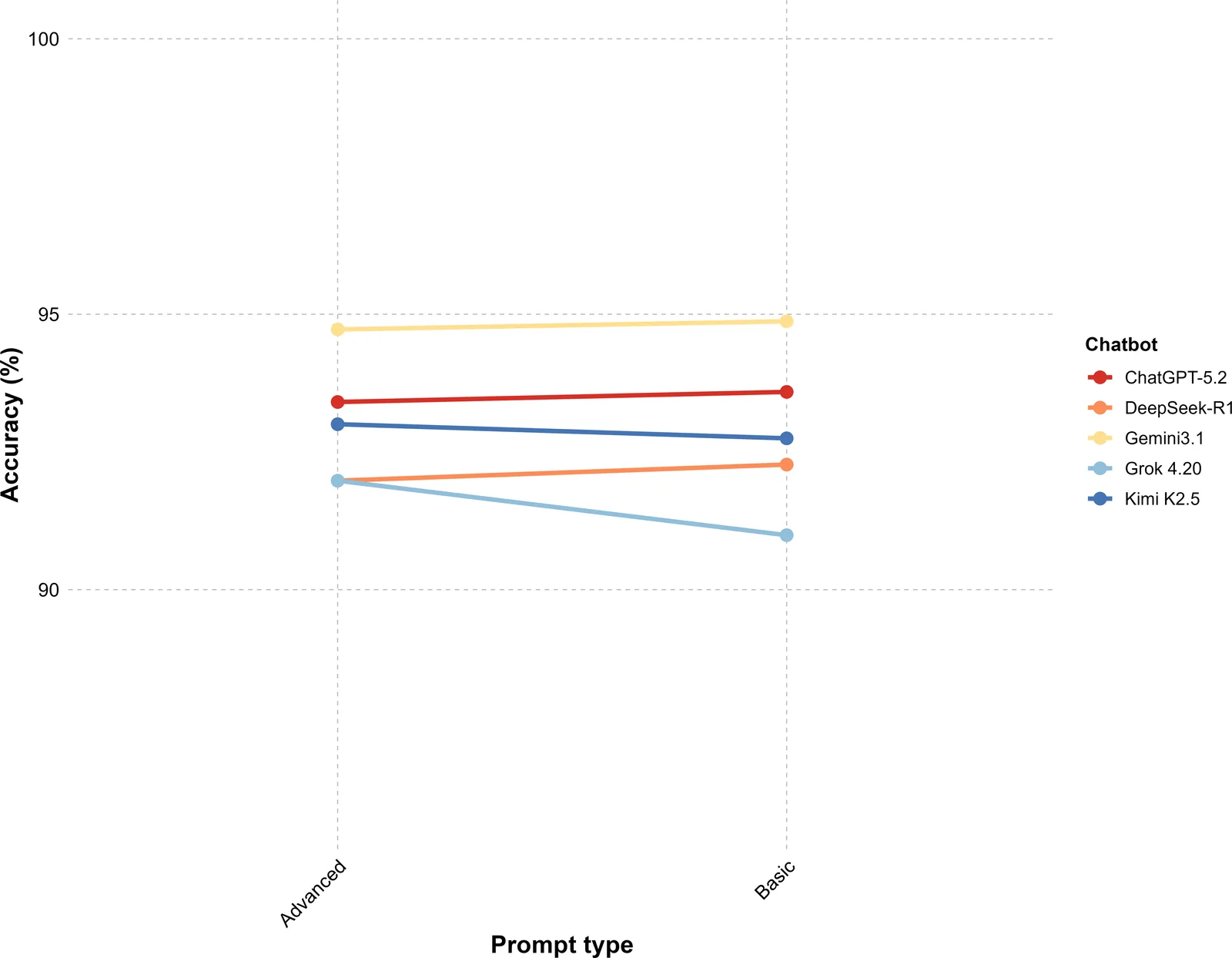
Chatbots’ accuracy in prompt level.

### Interaction Between Prompt Level and Question Type

The results of the analysis in Table 4 evaluate whether the complexity of the prompt level (advanced vs basic) influenced chatbot accuracy across diverse question formats. Across all five evaluated chatbots, the transition between prompt levels did not yield a statistically significant difference in performance for any question category (*P*>.05 for all 20 comparisons). Gemini 3.1 achieved the highest accuracy observed in the entire interaction analysis, correctly answering 621 of 630 MCQ responses (98.6%) under the basic prompt and 617 of 630 (97.9%) under the advanced prompt (*P*=.39), with similarly high and stable performance on yes or no questions (612 of 630, 97.1% advanced; 614 of 630, 97.5% basic; *P*=.73). DeepSeek-R1 showed the most complete stability across levels, returning identical accuracy rates for both clinical scenario questions (587 of 630, 93.2% under both advanced and basic; *P*>.99) and yes or no questions (585 of 630, 92.9% under both conditions; *P*>.99). Although the aggregate data showed relative stability overall, some chatbots exhibited numerical, nonsignificant declines under basic prompting. Grok 4.20 showed the largest such decline, falling from 581 of 630 (92.2%) correct yes or no responses under advanced prompt to 566 of 630 (89.8%) under basic, though this difference did not reach statistical significance (*P*=.14). Similarly, Kimi K2.5 showed a numerical decrease in clinical scenario performance, from 578 of 630 (91.7%) correct responses under advanced prompt to 565 of 630 (89.7%) under basic prompt, which likewise did not reach statistical significance (*P*=.21). ChatGPT-5.2 remained consistent across its largest question category, WH questions, with 761 of 840 (90.6%) correct under advanced prompt and 759 of 840 (90.4%) under basic prompt (*P*=.87). Taken together, these findings indicate that prompt level complexity does not meaningfully alter chatbot accuracy for any question format, reinforcing the overall prompt level result reported in Table 3.

**Table 4.** Interaction between prompt level and question type.

Chatbot and answers	Clinical scenario			MCQs^a^			WH Questions			Yes or no		
	Advanced	Basic	*P* value	Advanced	Basic	*P* value	Advanced	Basic	*P* value	Advanced	Basic	*P* value
ChatGPT-5.2			.76			.59			.87			.37
Correct	581 (92.2)	578 (91.7)		613 (97.3)	616 (97.8)		761 (90.6)	759 (90.4)		595 (94.4)	602 (95.6)	
Incorrect	49 (7.8)	52 (8.3)		17 (2.7)	14 (2.2)		79 (9.4)	81 (9.6)		35 (5.6)	28 (4.4)	
DeepSeek-R1			>.99			.44			.94			>.99
Correct	587 (93.2)	587 (93.2)		583 (92.5)	590 (93.7)		756 (90.0)	757 (90.1)		585 (92.9)	585 (92.9)	
Incorrect	43 (6.8)	43 (6.8)		47 (7.5)	40 (6.3)		84 (10)	83 (9.9)		45 (7.1)	45 (7.1)	
Gemini 3.1			.41			.39			.67			.73
Correct	595 (94.4)	588 (93.3)		617 (97.9)	621 (98.6)		762 (90.7)	767 (91.3)		612 (97.1)	614 (97.5)	
Incorrect	35 (5.6)	42 (6.7)		13 (2.1)	9 (1.4)		78 (9.3)	73 (8.7)		18 (2.9)	16 (2.5)	
Grok 4.20			.61			.58			.92			.14
Correct	581 (92.2)	576 (91.4)		566 (89.8)	560 (88.9)		783 (93.2)	782 (93.1)		581 (92.2)	566 (89.8)	
Incorrect	49 (7.8)	54 (8.6)		64 (10.2)	70 (11.1)		57 (6.8)	58 (6.9)		49 (7.8)	64 (10.2)	
Kimi K2.5			.21			.82			.51			.90
Correct	578 (91.7)	565 (89.7)		589 (93.5)	587 (93.2)		776 (92.4)	783 (93.2)		596 (94.6)	597 (94.8)	
Incorrect	52 (8.3)	65 (10.3)		41 (6.5)	43 (6.8)		64 (7.6)	57 (6.8)		34 (5.4)	33 (5.2)	

a MCQ: multiple-choice question.

### Impact of Question Type on Chatbot Reliability

Table 5 and Figure 5 present the distribution of chatbot responses across various question types, revealing that every evaluated chatbot model was significantly influenced by the question format. Gemini 3.1 demonstrated highly significant variability (*P*<.001), reaching near-peak performance in MCQ with a (1238/1260, 98.3%) rate of accuracy and in yes or no questions at (1226/1260, 97.3%), while appearing more sensitive to the WH Question format at (1529/1680, 91%). Similarly, ChatGPT-5.2 performed exceptionally well in the MCQs, achieving 1229 of 1260 (97.5%) responses rate of accuracy, but exhibited a specific vulnerability in WH questions, where accuracy dropped to 1520 of 1680 (90.5%*; P*<.001). DeepSeek-R1 also showed highly significant variation across formats (*P*=.002), maintaining relatively high accuracy in clinical scenarios (1174/1260, 93.2%) but struggling with the consistency of WH questions (1513/1680, 90.1%). Grok 4.20 recorded significant inconsistency (*P*=.003), yielding its highest error rate in the MCQ questions (134/1260, 10.6%). In contrast to the other chatbot models, Kimi K2.5 maintained its highest accuracy in the yes or no question (1193/1260, 94.7%), while showing its lowest performance in clinical scenarios (1143/1260, 90.7%), though this fluctuation was still statistically significant (*P*=.001).

**Table 5.** Distribution of chatbot performance across question types.

Chatbot and answers		Clinical scenario, n (%)	MCQ^a^, n (%)	WH Question, n (%)	Yes or no, n (%)	*P* value
ChatGPT-5.2						<.001
Correct		1159 (92)	1229 (97.5)	1520 (90.5)	1197 (95)	
Incorrect		101 (8)	31 (3)	160 (9.5)	63 (5)	
DeepSeek-R1						.002
Correct		1174 (93.2)	1173 (93.1)	1513 (90.1)	1170 (92.9)	
Incorrect		86 (7)	87 (7)	167 (9.9)	90 (7)	
Gemini3.1						<.001
Correct		1183 (93.9)	1238 (98.3)	1529 (91)	1226 (97.3)	
Incorrect		77 (6)	22 (2)	151 (9)	34 (3)	
Grok 4.20						.003
Correct		1157 (91.8)	1126 (89.4)	1565 (93.2)	1147 (91)	
Incorrect		103 (8)	134 (10.6)	115 (6.8)	113 (9)	
Kimi K2.5						.001
Correct		1143 (90.7)	1176 (93.3)	1559 (92.8)	1193 (94.7)	
Incorrect		117 (9)	84 (7)	121 (7.2)	67 (5)	

a MCQ: multiple-choice question.

**Figure 5.**
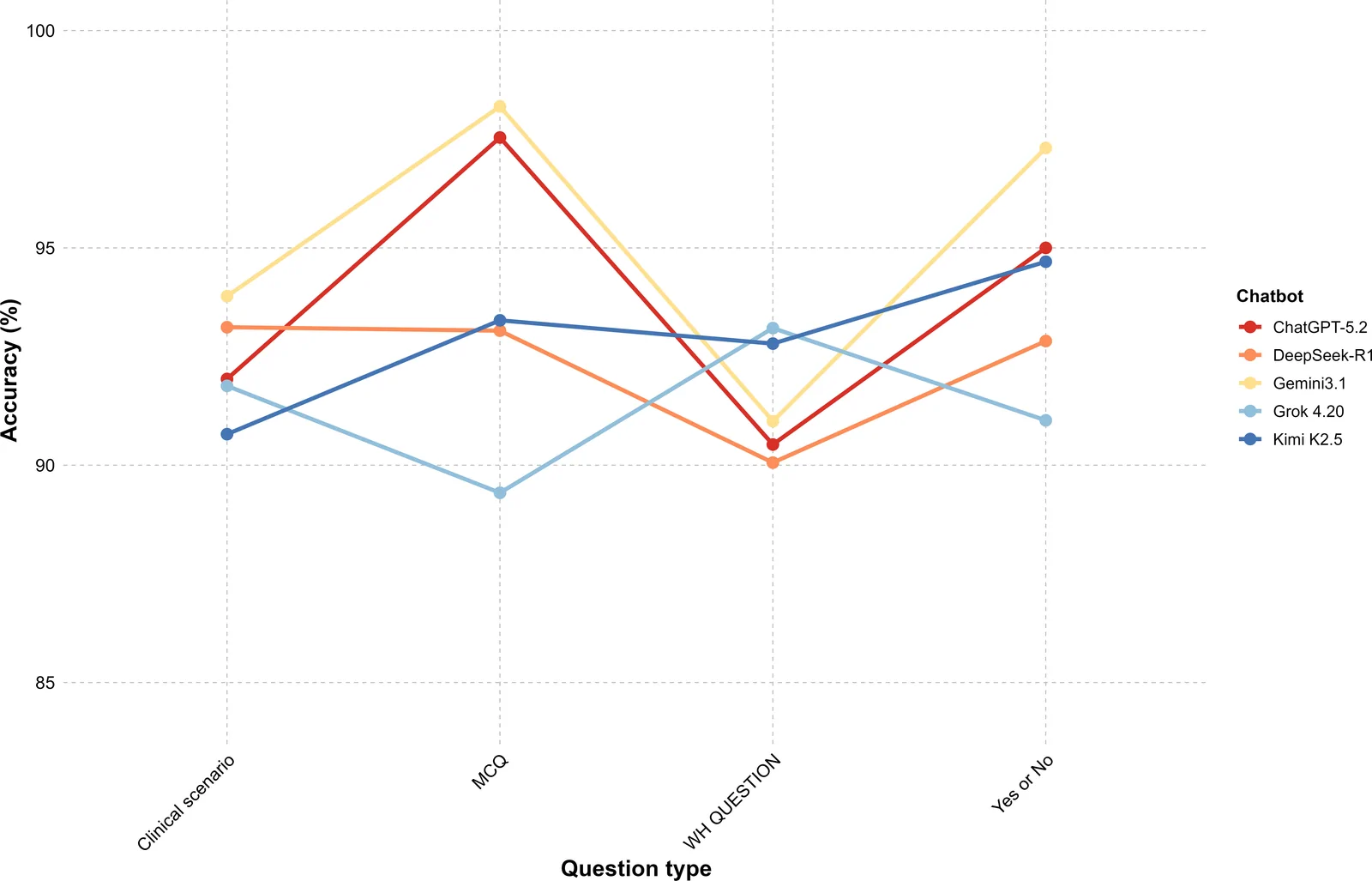
Distribution of chatbot accuracy through question type. MCQ: multiple-choice question.

### Impact of Language on Chatbot Accuracy

Table 6 details the performance of the evaluated chatbots across languages. Statistical analysis revealed that language was a significant predictor of accuracy for ChatGPT-5.2 and DeepSeek-R1 *P*<.001, while others maintained high linguistic stability (Figure 6). Gemini 3.1 demonstrated the highest overall consistency across English and Arabic 1741/1820, 95.7%, and 1711/1820, 94.0%, respectively, with no statistically significant variation (*P*=.08). Similarly, Grok 4.20 and Kimi K2.5 showed remarkable stability, with no significant differences in accuracy across all languages (*P*=.99 and *P*=.23, respectively). In contrast, ChatGPT-5.2 performed significantly better in Chinese, achieving (1734/1820, 95.3%) accuracy, but declined to (1676/1820, 92.1%) when processing English queries. Conversely, DeepSeek-R1 showed a strong preference for English, achieving (1725/1820, 94.8%) accuracy compared to 90.8% in both Arabic (1652/1820) and Chinese (1653/1820). These findings suggest that while AI chatbot models are generally capable across languages, certain AI chatbots like Gemini 3.1 are more linguistically robust, whereas others remain sensitive to the language of the clinical query.

**Table 6.** Distribution of chatbot performance across languages. (N=1820).

Chatbot and answers, n (%)	Arabic, n (%)	Chinese, n (%)	English, n (%)	*P* value
ChatGPT-5.2				<.001
Correct	1695 (93.1)	1734 (95.3)	1676 (92.1)	
Incorrect	125 (6.9)	86 (4.7)	144 (7.9)	
DeepSeek-R1				<.001
Correct	1652 (90.8)	1653 (90.8)	1725 (94.8)	
Incorrect	168 (9.2)	167 (9.2)	95 (5)	
Gemini 3.1				.08
Correct	1711 (94.0)	1724 (94.7)	1741 (95.7)	
Incorrect	109 (6.0)	96 (5)	79 (4)	
Grok 4.20				.99
Correct	1664 (91.4)	1666 (91.5)	1665 (91.5)	
Incorrect	156 (8.6)	154 (8.5)	155 (8.5)	
Kimi K2.5				.23
Correct	1687 (92.7)	1679 (92.3)	1705 (93.7)	
Incorrect	133 (7.3)	141 (7.7)	115 (6.3)	

**Figure 6.**
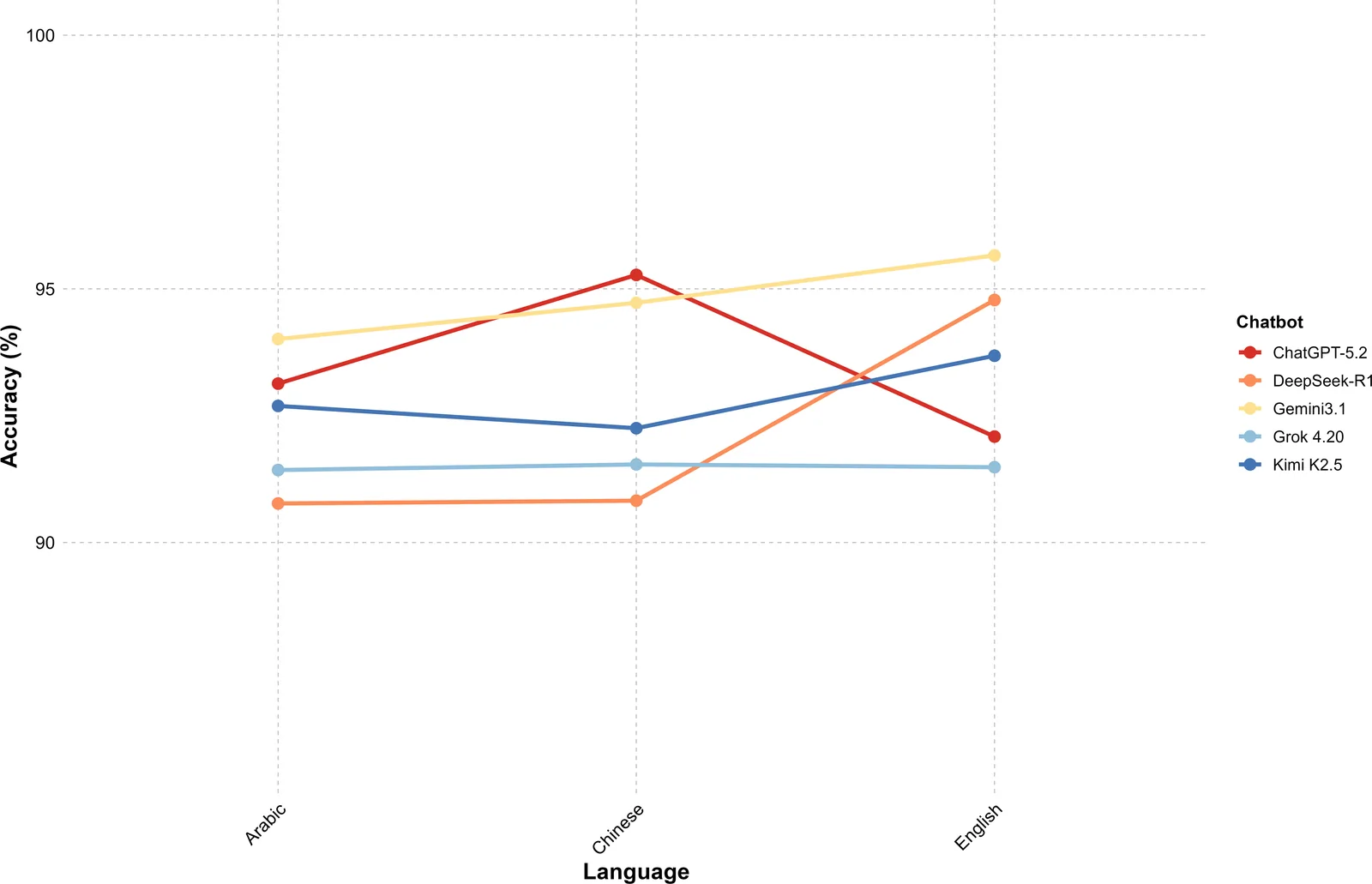
Chatbot accuracy distribution by language.

### Overall Accuracy of Chatbots

As shown in Table 7, the chatbot model was a highly significant predictor of accuracy (*χ*²_4_=54.66; *P*<.001). Post hoc pairwise comparisons of column proportions with Bonferroni correction showed that Gemini 3.1 (5176/5460, 94.8%) achieved significantly higher accuracy than other chatbot models. No other pairwise comparisons reached statistical significance after correction, indicating that ChatGPT-5.2, DeepSeek-R1, Kimi K2.5, and Grok 4.20 form a statistically comparable performance tier, except for the ChatGPT-5.2/Grok 4.20 contrast, while Gemini 3.1 stands as the single top-performing model.

**Table 7.** Pairwise post hoc comparison of chatbot model accuracy. a, b, c: each subscript letter denotes a subset of chatbot categories whose column proportions do not differ significantly from each other at the .05 level.

Answers	ChatGPT-5.2, n (%)	DeepSeek-R1, n (%)	Gemini3.1, n (%)	Grok 4.20, n (%)	Kimi K2.5, n (%)	Total, n (%)	*P* value^a^
Correct	5105_a_ (93.5)	5030_a,b_ (92.1)	5176_c_ (94.8)	4995_b_ (91.5)	5071_a,b_ (92.9)	25,377 (92.96)	<.001
Incorrect	355_a_ (6.5)	430_a,b_ (7.9)	284_c_ (5.2)	465_b_ (8.5)	389_a,b_ (7.1)	1923 (7)	<.001
Total	5460 (100.0)	5460 (100.0)	5460 (100.0)	5460 (100.0)	5460 (100.0)	27,300 (100.0)	<.001

a Pearson chi-square (*P*<.001).

Table 8 demonstrates that the overall accuracy across all chatbot responses was 25,377 of 27,300 (92.96%, 95% CI 92.65‐93.26). Among the evaluated models, Gemini 3.1 demonstrated the highest accuracy (5176/5460, 94.80%, 95% CI 94.21‐95.39), followed by ChatGPT-5.2 (5105/5460, 93.5%, 95% CI 92.85‐94.15). The remaining models showed slightly lower performance, including Kimi K2.5 (5071/5460, 92.9%), DeepSeek-R1 (5030/5460, 92.1%), and Grok 4.20 (4995/5460, 91.5%), as shown in Figure 7.

**Table 8.** Performance accuracy and 95% CI of evaluated chatbots.

Chatbot	Correct answers, n	Mean accuracy, %	95% CI
Gemini 3.1	5176	94.8	94.21‐95.39
ChatGPT-5.2	5105	93.5	92.85‐94.15
Kimi K2.5	5071	92.9	92.19‐93.56
DeepSeek-R1	5030	92.1	91.41‐92.84
Grok 4.20	4995	91.5	90.75‐92.21
Total	25,377	93.0	92.65‐93.26

**Figure 7.**
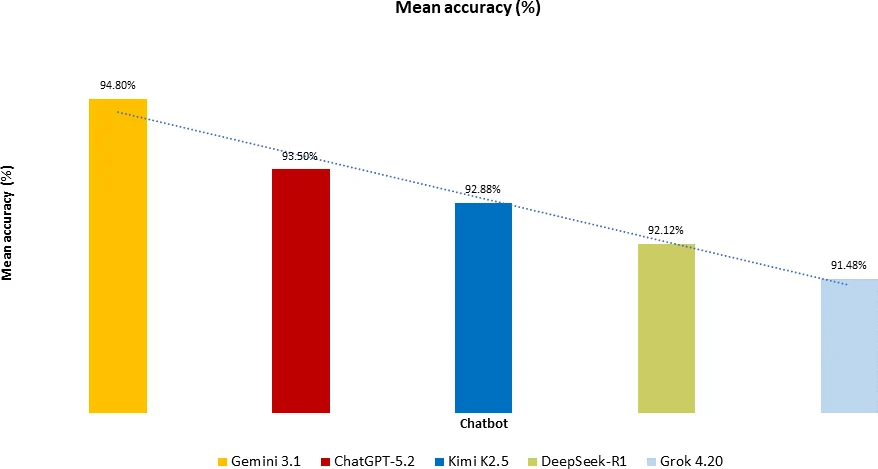
Overall mean accuracy among chatbots.

## Discussion

### Principal Findings

This study evaluated five publicly accessible, internet-based AI chatbots as potential digital health resources for dental medical emergency management—arguably one of the highest-stakes use cases for any consumer-facing health technology. The overarching finding is a paradox characteristic of the current digital health landscape: aggregate accuracy is high enough to inspire misplaced confidence (92.96%); yet, the residual error rate is clinically unacceptable in a domain where a single incorrect recommendation can precipitate irreversible patient harm. Our results demonstrate a high rate of accuracy across all 5 evaluated LLM-based chatbots, with a total mean accuracy of 92.96%. This high level of performance is in line with recent benchmarking studies, which similarly report that contemporary LLMs frequently attain accuracies exceeding 90% on high-stakes medical multiple-choice examinations. For example, a recent comparative analysis found that Gemini-family models reached the highest accuracy on a national licensing examination, while several competing models also surpassed the predefined passing threshold. Taken together, our findings confirm that current-generation LLMs possess the requisite baseline knowledge for high-level clinical information processing in structured testing environments [13,40]. Among these models, Gemini 3.1 achieved the highest overall accuracy. These findings align with emerging evidence from multiple dental AI studies. In orthodontic emergency scenarios, Gemini and ChatGPT-4.0 demonstrated superior accuracy compared to other models, with Gemini significantly outperforming competitors in fixed orthodontic treatment categories [41]. Similarly, in restorative dentistry specialty examination questions, Gemini Advanced achieved the highest accuracy (96.28%), followed closely by ChatGPT-4o Plus (93.62%) [42]. Additionally, findings align with a study by Zhang et al [43], which demonstrated that Gemini-3 achieved the highest overall accuracy among five frontier LLMs.

The second-tier performance of ChatGPT-5.2 (93.50%) and Kimi K2.5 (92.88%) in our study corresponds with literature reporting ChatGPT models as reliable performers. In dental trauma management, ChatGPT-4.0 achieved 95.6% accuracy for avulsion-related questions, demonstrating the highest consistency among evaluated models [44]. However, the same study noted that ChatGPT-3.5 showed weaker agreement, suggesting that newer iterations represent substantial improvements. Our findings with ChatGPT-5.2 extend this observation, confirming that advanced versions maintain high accuracy while showing specific strengths in structured question formats such as multiple-choice items (97.5%).

In this study, DeepSeek-R1 achieved an accuracy of 92.12%, whereas Grok 4.20 achieved 91.48%. DeepSeek’s performance in our study aligns with recent comparative analyses where DeepSeek demonstrated the lowest accuracy among advanced models in restorative dentistry examinations [42] and the slowest response times in pediatric dental trauma assessments [45]. Whereas a study conducted by Bashah et al [46] found that DeepSeek achieved the highest accuracy among comparative chatbots, Gemini, ChatGPT-4o, and Perplexity. Although the overall accuracy exceeded 90%, the presence of incorrect responses in approximately 7% of cases remains clinically significant, as even a single incorrect recommendation during a medical emergency could potentially compromise patient safety.

While many studies focus on performance changes over time, several specific research efforts have found no significant difference in chatbot performance when tested over shorter intervals, such as days or weeks [47,48], similar to our finding that indicated no significant variation in accuracy rates over time. Research evaluating LLMs in clinical decision-making demonstrated that prompt engineering does not universally improve model performance and may even reduce accuracy for certain clinical tasks [49,50]. These findings indicate that, particularly for newer models, the level of prompt complexity, basic versus advanced prompting, may have minimal influence on overall response quality, similar to this study, which shows that the lack of significant variance across chatbots indicates that the level of prompt complexity does not meaningfully impact the reliability of these chatbots in clinical knowledge assessment.

The current findings are consistent with previous studies demonstrating that the format of a question significantly influences the performance of LLM-based chatbots. Prior research comparing multiple-choice and free-response medical questions reported a substantial deterioration in accuracy when models answered open-ended questions instead of MCQs. Similarly, evaluations of AI chatbots using medical examination datasets have shown that performance varies across question formats, with structured questions such as MCQs generally yielding higher accuracy than other formats. These studies confirm a significant association between question type and model accuracy [51-53]. Our observation that ChatGPT-5.2 excelled in MCQs (97.5%) while Kimi K2.5 appeared more sensitive to clinical scenarios aligns with published research. In dental trauma education, chatbots showed significantly higher correct response rates for multiple-choice and fill-in-the-blank questions compared to dichotomous questions [54].

Our findings also demonstrated a statistically significant association between the language of the query and chatbot accuracy. Although the differences were relatively small, English questions showed slightly higher accuracy than Arabic and Chinese queries. This pattern of language-based performance variation is consistent with emerging evidence in the dental and medical AI literature. In a study evaluating LLMs for mild cognitive impairment management, English responses were significantly more comprehensible and specific than Chinese responses across ChatGPT-4o, Gemini, and Kimi models (*P*<.001). The authors attributed this disparity to training data distributions, noting that models like Gemini are optimized primarily for English, with corpora focusing on English-language data for greater contextual precision, limiting their ability to handle other languages like Chinese effectively. Conversely, Kimi—designed specifically for Chinese language users with a specialized dataset tailored to Chinese discourse—demonstrated that monolingual focus can enhance performance in specific populations [55]. Similarly, a comparative study of ChatGPT-4.5 and DeepSeek-R1 in answering orthognathic surgery questions found that English responses significantly outperformed Turkish responses in readability, word count, and accuracy (*P*<.05). The authors emphasized that while both models effectively generated clinically relevant responses, the superior performance of English responses underscores the need for further multilingual optimization [56]. Additional observations have been reported in previous multilingual evaluations of LLMs. For example, a study comparing chatbot responses to cardiovascular-prevention questions in English and Chinese found that ChatGPT-4 generated more accurate responses for English prompts than for Chinese prompts [57]. Additionally, a study evaluating LLM performance on the Chinese National Medical Licensing Examination highlighted that most models are trained primarily on English-language corpora, which can influence performance when responding to non-English queries [58]. A cross-lingual clinical study conducted by Niu et al [59], to evaluate LLM responses to ophthalmology questions also reported performance differences across languages, with some models performing better in English and others showing strengths in Chinese clarity depending on training data and linguistic characteristics. Interestingly, a study on maxillofacial prosthetics questions found no statistically significant differences between English and Turkish performance across ChatGPT-4o, Gemini 2.5 Flash, DeepSeek V3, and Claude Sonnet 4, with strong positive correlations between languages [60]. These findings suggest that differences in training data distribution, linguistic structure [61], and translation processes [62] may contribute to variations in chatbot accuracy across languages. Consequently, although modern AI chatbots demonstrate strong multilingual capabilities, their performance may still vary depending on the language in which questions are posed. For medical emergency contexts in multilingual populations, these findings underscore the importance of validating chatbot performance in the languages they will encounter clinically and considering language-specific limitations when implementing AI tools in diverse health care settings.

The findings of this study carry direct implications for digital health governance. Current regulatory frameworks for AI in health care—including guidance from the FDA, the EU AI Act, and equivalent bodies—largely address AI embedded within regulated medical devices or electronic health records. Publicly accessible chatbots that are used as informal clinical decision-support tools exist in a governance gap. Our data suggest that these tools perform well enough that clinicians may not recognize when they have received an incorrect recommendation—a phenomenon that is more dangerous than obvious failure. We propose that professional dental and medical associations should issue explicit guidance on the appropriate and inappropriate uses of AI chatbots in clinical settings, that future evaluations adopt safety-weighted rather than binary accuracy scoring, and that multilingual validation be made a prerequisite for any AI tool intended for use in diverse clinical populations.

This study, like others, has several limitations. First, the study evaluated only 5 contemporary AI chatbots. Second, these chatbots were evaluated as available at the time of testing. Third, while multiple-choice and yes or no formats are unlikely to reflect how a clinician would phrase a question during an actual emergency—since such structured options would not be preformulated in real time—these formats were retained as a knowledge-benchmarking control condition rather than a simulation of authentic emergency use; free-text WH questions and clinical scenarios were included specifically to capture the more realistic query format. Future multilingual emergency management assessments should include addtional chatbots , such as Claude, for a more comprehensive cross-platform comparison. Additionally, the 5 chatbots were tested under their most accessible free-tier default rather than a matched reasoning condition, which may lead to performance differences and should be addressed in future work with reasoning equality designs.

The field of LLMs is evolving rapidly, and future model updates or new systems may demonstrate different levels of performance. Consequently, the results should be interpreted as a snapshot of current AI capabilities rather than a permanent benchmark. Although the study incorporated three languages (English, Arabic, and Chinese) to evaluate multilingual performance, translation procedures may still introduce subtle linguistic variations that influence chatbot responses. Differences in training data distribution across languages may also affect model outputs. Responses were categorized using a binary correct and incorrect scoring system, which simplifies evaluation but does not capture partial correctness, clinical reasoning quality, or safety of recommendations. Some responses may have been technically correct but clinically incomplete. Finally, the study assessed chatbot accuracy across 7 days, which provides short-term temporal reliability but does not reflect potential changes in model performance over longer periods or after system updates.

Future research should investigate chatbot performance through prospective simulations, clinician-AI interaction models, and real clinical workflows, and evaluate the clinical safety, reasoning transparency, and potential risks of AI-generated medical guidance in emergency settings.

### Conclusion

In conclusion, contemporary LLM-based chatbots demonstrated high overall accuracy in answering questions related to medical emergency management in dental practice. Among the evaluated AI Chatbot models, Gemini 3.1 achieved the highest overall performance and the greatest stability across languages and prompt conditions, followed by ChatGPT-5.2 and Kimi K2.5. Despite this high level of performance, chatbot accuracy varied significantly according to question format and language, with structured formats such as MCQs yielding higher accuracy than open-ended questions. Prompt complexity and testing time did not significantly influence chatbot performance, indicating stable outputs across different prompting strategies and short-term testing periods. Although these findings suggest that modern AI chatbots possess substantial medical knowledge and may serve as supportive educational or informational tools, their use as real-time clinical decision support during dental medical emergencies remains inappropriate and potentially hazardous due to the possibility of incorrect or incomplete recommendations. Further research is needed to evaluate clinical safety, decision-making reliability, and integration strategies before AI chatbots can be considered for responsible implementation in emergency health care environments. Given that these tools are freely accessible to any clinician or patient with an internet connection, their effective governance represents an urgent priority for the digital health and health informatics communities.

## Supplementary material

10.2196/96731 Multimedia Appendix 1 English version of the questions.

## Abbreviations

BLS: basic life support
GenAI: generative AI
LLM: large language model
MCQ: multiple-choice question
ML: machine learning
