# Supplementary material for: Evaluating Internet-Based AI Chatbots as Unregulated Digital Health Resources in Dental Emergency Management: Comparative Multilingual Study
Source: JMIR Med Inform. 2026 Oct 27;14:e96731. doi: 10.2196/96731 (PMC13712258; doi:10.2196/96731)
Supplement: Supplementary file 1 [file medinform-v14-e96731-s001.pdf]

Supplemental File 1

Questions

Multiple Choice Questions (MCQ)

1. What is the most commonly reported emergency in the dental office, accounting for over 50% of all reported cases?
 A. Myocardial infarction
 B. Vasodepressor syncope (common faint)
 C. Anaphylaxis
 D. Insulin shock
2. According to Matsuura, what percentage of life-threatening systemic complications in the dental office developed during or immediately following local anesthetic administration?
 A. 15.2%
 B. 38.9%
 C. 54.9%
 D. 77.8%
3. In the basic management algorithm for all medical emergencies, what does the 'D' stand for?
 A. Diagnosis
 B. Definitive care (Diagnosis, Drugs, Defibrillation)
 C. Dental treatment
 D. Danger assessment
4. What is the recommended dose of epinephrine (1:1,000) for an adult experiencing anaphylaxis?
 A. 0.1 mg
 B. 0.3 mg
 C. 1.0 mg
 D. 5.0
5. Which team member is responsible for staying with the victim and administering Basic Life Support (BLS) as required?
 A. Member 1
 B. Member 2
 C. Member 3
 D. The receptionist
6. What is the minimum standard for oxygen cylinders in a dental office emergency kit?
A. One "A" cylinder
B. One "D" cylinder
C. One "E" cylinder
D. One "H" cylinder
7. Nitroglycerin tablets are known to degrade and become ineffective after being exposed to air for approximately how long?
A. 24 hours
B. 1 week
C. 12 weeks
D. 1 year

8. Which drug is the primary choice for managing a conscious patient experiencing a bronchospasm (asthma attack)?
A. Epinephrine
B. Albuterol (Salbutamol)
C. Diphenhydramine
D. Hydrocortisone
9. Status epilepticus is defined as a life-threatening situation where a seizure lasts more than:
A. 30 seconds
B. 1 minute
C. 5 minutes
D. 10 minutes
10. What is the ratio of chest compressions to ventilations for an adult victim in cardiac arrest?
A. 15:2
B. 20:1
C. 30:2
D. 50:2
11. The most common allergen in the dental environment today is:
A. Penicillin
B. Aspirin
C. Latex
D. Lidocaine
12. In the case of an unconscious patient, how should they be positioned?
A. Upright
B. Supine with feet elevated
C. Trendelenburg
D. Prone
13. Which drug is indicated for a mild (non-life-threatening) allergic reaction?
A. Epinephrine
B. Diphenhydramine (Benadryl)
C. Albuterol
D. Glucagon
14. Myocardial infarction (MI) deaths most often occur within the first two hours and are due to:
A. Respiratory arrest
B. Ventricular dysrhythmias (fibrillation)
C. Blood loss
D. Insulin shock
15. What is the target depth for chest compressions on an adult?
A. 1 cm
B. 3 cm
C. At least 5 cm (2 inches)
D. 10 cm
16. Which medical emergency results from a sudden decrease in cerebral oxygenation and perfusion?
A. Hypertension
B. Syncope
C. Seizure

D. Hyperventilation
17. If a patient has previous history of angina but complains of crushing chest pain, the situation should be treated as:
A. Anxiety
B. Heartburn
C. Acute Myocardial Infarction (AMI)
D. Syncope
18. What is the drug of choice for managing a patient in severe hypoglycemia who is unconscious?
A. Oral orange juice
B. Glucagon (IM)
C. Insulin
D. Nitroglycerin
19. Which piece of equipment is essential for retrieving small objects from the oral cavity or pharynx?
A. Laryngoscope
B. Magill intubation forceps
C. Scalpel
D. Suction tip
20. A patient with "incapacitating systemic disease that is a constant threat to life" is classified as ASA Status:
A. ASA 1
B. ASA 2
C. ASA 3
D. ASA 4
21. Diabetic ketoacidosis is characterized by breath that smells:
A. Like alcohol
B. Fruity or sweet (acetone)
C. Like tobacco
D. Foul and metallic
22. "SBAR" communication stands for:
A. Status, Background, Airway, Rescue
B. Situation, Background, Assessment, Recommendation
C. Symptoms, Body-temp, Airway, Response
D. Speed, Breathing, Assessment, Recovery
23. Hyperventilation typically causes which chemical imbalance in the blood?
A. Respiratory acidosis
B. Respiratory alkalosis
C. Metabolic acidosis
D. Hyperkalemia
24. The recommended concentration for IM epinephrine is:
A. 1:100
B. 1:1,000
C. 1:10,000
D. 1:100,000
25. A patient exhibiting "white-knuckle syndrome" is showing signs of: A. Anaphylaxis B. Moderate anxiety C. Cardiac arrest D. Stroke Correct Answer: B. Moderate anxiety.
26. How often should unannounced "emergency drills" be conducted in the dental office?

A. Annually
B. Every 6 months
C. Every 3 months
D. Monthly
27. Which condition involves the sudden onset of facial weakness, arm weakness, and speech problems?
A. Hypoglycemia
B. Stroke (CVA)
C. Angina
D. Seizure
28. For a patient experiencing an anginal attack, how many doses of nitroglycerin can be given before activating EMS (assuming pain persists)?
A. One
B. Two
C. Three
D. Five
29. Aspirin should be administered in a suspected MI to:
A. Relieve pain
B. Inhibit platelet aggregation
C. Lower blood pressure
D. Stop a seizure
30. Which of the following is T a component of the primary BLS survey?
A. Airway
B. Breathing
C. Circulation
D. Drug administration

Yes/ No Questions

31. Is vasovagal syncope the most common medical emergency in the dental office?
32. Should Member 1 of the emergency team leave the victim to find Member 2?
33. Is oxygen considered the most useful drug in the entire emergency kit?
34. Is it recommended to use a "stereotyped" or "standardized" commercial drug kit for every practice?
35. Does anxiety increase the risk of an emergency occurring during dental treatment?
36. Is 911 the universal emergency number for EMS in California?
37. Can a patient with a known history of stable angina be treated as ASA class 3?
38. Should a dentist administer drugs that they are not trained to use
39. Is epinephrine mandatory for the survival of a patient in anaphylaxis?
40. Is a supine position recommended for a conscious patient experiencing respiratory distress?
41. Should dental care be terminated immediately once an emergency is recognized?
42. Is it safe to place objects into the mouth of a seizing patient?
43. Is hypoglycemia most common in Type 1 diabetic patients who skip a meal?
44. Can hyperventilation lead to convulsions if prolonged?
45. Should a dentist wait for absolute certainty of the emergency nature before calling EMS?
46. Is the "E" cylinder the minimum size recommended for oxygen storage?
47. Is status epilepticus considered a life-threatening situation?

48. Does the GDC require all registrants to be trained in dealing with medical emergencies?
49. Is aspirin used to manage pain during a myocardial infarction in the dental office?
50. Should a patient who has just recovered from syncope be allowed to drive themselves home?
51. Is epinephrine (1:1,000) recommended for IV administration?
52. Can a dental nurse be responsible for recording a patient's vital signs?
53. Is it necessary to check emergency drug expiry dates every week?
54. Is a patient with a blood pressure of 200/115 classified as ASA 4?
55. Is it generally safe to treat a patient who has had a myocardial infarction within the last 3 months? (Strongly recommended to wait at least 6 months).
56. Does "KISS" stand for "Keep It Simple, Stupid"?
57. Is albuterol used as a vasodilator for chest pain?
58. Should Member 2 of the team bring the AED to the scene?
59. Is the pulse check during BLS performed at the radial artery for adults?
60. Can syncope be triggered by the sight of a needle?

Short Answer Questions

61. Define a medical emergency in a dental practice.
62. What are the five categories used to recognize and manage emergencies?
63. What are the four steps of Basic Life Support (BLS)?
64. Where should the "Medical Emergencies" poster be placed?
65. What is the duty of Team Member 2 on hearing an emergency call?
66. List three clinical signs of a mild allergic reaction?
67. What is the most important drug in the emergency kit for a patient in anaphylactic shock?
68. Describe the management of a conscious patient experiencing hyperventilation?
69. How should an unconscious patient's airway be opened?
70. What is the objective of the "E" in the ABCDE mnemonic?
71. What is the treatment of choice for a conscious hypoglycemic patient?
72. Why should Member 3 meet the rescue team at the building entrance
73. What are the clinical signs of a stroke (CVA)?
74. What is the "White-knuckle syndrome"?
75. What is the recommended chest compression rate for adult CPR?
76. List three causes of respiratory distress.
77. What should be checked weekly in the emergency kit?
78. Define orthostatic (postural) hypotension?
79. What is the primary role of Team Member 3?
80. Name the specific drug used to reverse an opioid overdose?
81. What is the recommended needle length for IM injections in adults to ensure muscle penetration?
82. What is the significance of an ASA 1 patient?
83. Describe the "Kussmaul's respirations" associated with diabetic coma?
84. What is a "Transient Ischemic Attack" (TIA)?
85. What are the signs of a local anesthetic overdose?
86. What piece of equipment allows delivery of 100% oxygen?
87. How long should you palpate the carotid pulse during a BLS survey?
88. What is the "KISS" principle in emergency kit preparation?
89. Why is a thorough medical history check a form of risk management?

90. What drug category is Midazolam?
91. What is the most practical location for Team Member 3 during an emergency?
92. List two stress-induced emergencies?
93. What is the first thing a dentist should do when a patient feels "faint"?
94. What is the recommended dose for buccal Midazolam in a child aged 10-18?
95. Describe the "clonic phase" of a seizure?
96. What is the primary focus of prehospital management for a stroke victim?
97. Why is nitroglycerin spray sometimes preferred over tablets?
98. What is the purpose of an automated external defibrillator (AED)?
99. How many people, at minimum, should be present in a dental office during treatment?
100. What is the ultimate responsibility of the dentist during an emergency?
…………………………………………………………………………………………………

Clinical Scenarios Questions

I. Cardiovascular Emergencies

101. Scenario: A 55-year-old male with a history of stable angina experiences chest pain during an injection. What is the immediate management?
102. Scenario: A patient with no history of heart disease experiences crushing chest pain that does not resolve with rest. What is the presumptive diagnosis?
103. Scenario: You suspect a patient is having a myocardial infarction. What is the recommended aspirin protocol?
104. Scenario: A patient becomes unconscious, and you can't palpate a carotid pulse. What is the first action?
105. Scenario: What is the correct compression-to-ventilation ratio for adult CPR in the dental chair?
106. Scenario: A patient reports chest pain that was relieved by nitroglycerin but has not returned. How is this managed?

II. Allergic Reactions and Anaphylaxis

107. Scenario: A patient exhibits a rapid onset of hives, wheezing, and a drop in blood pressure (80/40) following a local anesthetic. What is the drug of choice? Epinephrine (Adrenalin)?
108. Scenario: What is the standard adult dose and route for epinephrine in anaphylaxis?
109. Scenario: Where is the most effective site for an emergency IM injection of epinephrine?
110. Scenario: How should you manage a mild, delayed allergic reaction involving only skin eruptions (urticaria?
111. Scenario: A patient with anaphylaxis does not show improvement after the first dose of adrenaline. When can you repeat the dose?

III. Airway and Breathing Crises

112. Scenario: A known asthmatic patient begins wheezing and using accessory muscles to breathe. What is the first-line treatment?
113. Scenario: An asthmatic patient's distress continues despite using a bronchodilator. What is the next step?

114. Scenario: A patient is lightheaded, breathing rapidly, and complains of tingling in their fingers (paresthesia). How do you treat this?
115. Scenario: An object is dropped into the oropharynx, and the patient is coughing forcefully. How should you intervene?
116. Scenario: A patient who was choking becomes unconscious. What is the immediate priority?
117. Scenario: Which piece of equipment is specifically used to retrieve small objects from the oral cavity or pharynx? Magill intubation forceps.
IV. Neurologic Events (Stroke and Seizures)
118. Scenario: A patient exhibits sudden facial weakness, arm weakness on one side, and slurred speech. What is the priority?
119. Scenario: A patient has a generalized tonic-clonic seizure in the dental chair. How do you protect them?
120. Scenario: A seizure lasts longer than 5 minutes. What is this condition, and how is it managed
121. Scenario: How should a patient be positioned once a seizure has ended?

V. Metabolic and Other Emergencies

122. Scenario: A Type 1 diabetic patient who skipped breakfast becomes disoriented and sweaty. How do you treat them?
123. Scenario: A hypoglycemic patient loses consciousness. What is the definitive management?
124. Scenario: A patient on long-term corticosteroid therapy collapses with low blood pressure and a rapid, weak pulse. What is suspected? Acute adrenal insufficiency (Adrenal Crisis).
125. Scenario: A patient feels faint and loses consciousness after an injection. What is the most likely cause and first step?
126. Scenario: A patient experiences a sudden drop in blood pressure when the dental chair is returned to an upright position. What is this? Orthostatic (postural) hypotension.
127. Scenario: What are the signs of a local anesthetic overdose in a child?
128. Scenario: A pregnant patient feels dizzy and faint while lying supine. How do you reposition her?
129. Scenario: What is the "cardiac dose" of epinephrine for a patient with stable angina (ASA 3)?
130. Scenario: Which communication tool is recommended to ensure effective reporting when calling EMS?))
